# Nanostructured Dihydroartemisinin Plus Epirubicin Liposomes Enhance Treatment Efficacy of Breast Cancer by Inducing Autophagy and Apoptosis

**DOI:** 10.3390/nano8100804

**Published:** 2018-10-09

**Authors:** Ying-Jie Hu, Jing-Ying Zhang, Qian Luo, Jia-Rui Xu, Yan Yan, Li-Min Mu, Jing Bai, Wan-Liang Lu

**Affiliations:** State Key Laboratory of Natural and Biomimetic Drugs, Beijing Key Laboratory of Molecular Pharmaceutics and New Drug System, School of Pharmaceutical Sciences, Peking University, Beijing 100191, China; yingjie.hu93@gmail.com (Y.-J.H.); zhangjingying1995@126.com (J.-Y.Z.); Luoqian7777@126.com (Q.L.); xujiarui228@foxmail.com (J.-R.X.); yanyan1992111@163.com (Y.Y.); liminmu@163.com (L.-M.M.); vivalajing@163.com (J.B.)

**Keywords:** dihydroartemisinin, liposomes, autophagy, apoptosis, breast cancer

## Abstract

The heterogeneity of breast cancer and the development of drug resistance are the relapse reasons of disease after chemotherapy. To address this issue, a combined therapeutic strategy was developed by building the nanostructured dihydroartemisinin plus epirubicin liposomes. Investigations were performed on human breast cancer cells in vitro and xenografts in nude mice. The results indicated that dihydroartemisinin could significantly enhance the efficacy of epirubicin in killing different breast cancer cells in vitro and in vivo. We found that the combined use of dihydroartemisinin with epirubicin could efficiently inhibit the activity of Bcl-2, facilitate release of Beclin 1, and further activate Bax. Besides, Bax activated apoptosis which led to the type I programmed death of breast cancer cells while Beclin 1 initiated the excessive autophagy that resulted in the type II programmed death of breast cancer cells. In addition, the nanostructured dihydroartemisinin plus epirubicin liposomes prolonged circulation of drugs, and were beneficial for simultaneously delivering drugs into breast cancer tissues. Hence, the nanostructured dihydroartemisinin plus epirubicin liposomes could provide a new therapeutic strategy for treatment of breast cancer.

## 1. Introduction

Breast cancer is the most common malignancy and the leading cause of cancer-related mortality among women worldwide. In 2012 alone, there were an estimated 1.7 million cases diagnosed, and 521,900 breast cancer-related deaths [1]. The high morbidity of breast cancer is associated with the genetic heterogeneity of tumors [2]. In the last few decades, five different molecular subtypes of breast cancer have been identified, termed luminal A, luminal B, HER2-enriched, basal-like, and claudin-low [3]. Each subtype displays varying sensitivity to different cancer drugs [4]. If a universal drug formulation could treat varying subtypes of breast cancer cells, it would be useful for improving clinical treatment.

One emerging therapeutic strategy to overcome this obstacle has focused on drugs that preferentially induce autophagy and apoptosis in otherwise resistant cancer cells [5]. Induction of apoptosis is used as a cancer treatment strategy by triggering type I programmed death of refractory cancer cells. This is typically achieved through a combinational chemotherapy treatment. However, this approach tends to be ineffective in the case of heterogeneous breast cancers. In contrast, accumulating evidence suggests that autophagy, which triggers type II programmed cell death, could significantly enhance the efficacy of cancer treatments [6]. Autophagy (or “self-eating”) is a stress-induced process involving lysosomal degradation, which conserves cellular energy and maintains cytoplasmic homeostasis by eliminating protein aggregates and damaged organelles [7]. Autophagy is initiated by double-membraned autophagosomes that engulf portions of the cytoplasm. These autophagosomes ultimately fuse with lysosomes where the cytoplasmic contents are degraded [8,9].

Interestingly, autophagy plays a paradoxical role in cancer cells, and it can either promote or inhibit tumor formation depending upon the circumstances [10,11,12,13]. For example, defects in autophagy can lead to chronic tissue damage and inflammation, which may create an environment that promotes tumorigenesis. Moderate induction of autophagy also promotes the growth of cancer cells by limiting the stress response and supporting the metabolism and survival of cancer cells [14]. In contrast, excessive autophagy can also trigger type II programmed death in cancer cells due to the overconsumption of cytoplasmic proteins and organelles.

The relationship between these two types of programmed cell death is complicated. On one hand, apoptosis can lead to the activation or suppression of autophagy [15,16]. On the other hand, inhibition of apoptosis can result in either suppression or activation of autophagy [15,17]. The exact mechanisms underpinning these contradictory interactions remain unclear. Therefore, exploring the interaction between autophagy and apoptosis is an important avenue of research for developing treatments for heterogeneous breast cancers.

Dihydroartemisinin is a derivative of artemisinin, which is a naturally occurring sesquiterpene extracted from the traditional Chinese medicinal herb Qing Hao (*Artemesia annua*) [18]. Because the chemical structure has been optimized by modifying carbonyl groups into hydroxyl groups, dihydroartemisinin exhibits stronger antimalarial effect. Hence, it has become one of the first alternative antimalarial drugs recommended by the World Health Organization in the case where *Plasmodium falciparum* is resistant to traditional therapy. Further studies showed that dihydroartemisinin also exhibits strong antibacterial, antiviral, and anticancer activities [19,20,21,22]. In particular, dihydroartemisinin enables the induction of apoptosis in cancer cells through different mechanisms, such as by targeting JAK2/STAT3, NF-kB, JNK1/2, and p38 MAPK signaling pathways [23,24,25]. Besides, dihydroartemisinin also induces autophagy in ovarian cancer cells, pancreatic cancer cells, gliomas, and breast cancer cells [26,27,28,29]. Therefore, dihydroartemisinin was included in this study as a potential anticancer enhancer and epirubicin was used as an anticancer agent. Because drugs can distribute differentially in tissues, the two agents were incorporated into liposome vesicles to simultaneously deliver both drugs to cancer cells. The efficacy of this treatment approach was examined both in vitro (in human breast cancer cell lines) and in vivo (using mouse xenografts).

## 2. Materials and Methods

### 2.1. Preparation of Liposomes

Three types of liposomes were fabricated, including dihydroartemisinin plus epirubicin liposomes, dihydroartemisinin only liposomes, and epirubicin only liposomes.

To construct dihydroartemisinin liposomes, egg phosphatidylcholine (EPC), cholesterol, polyethylene glycol-distearoylphosphosphatidylethanolamine (PEG_2000_-DSPE, NOF Corporation, Tokyo, Japan) (EPC:CHOL:PEG_2000_-DSPE = 65:30:5, µmol/µmol), and dihydroartemisinin (Tokyo Chemical Industry Co., Ltd., Tokyo, Japan) were dissolved in chloroform in a pear-shaped flask. Chloroform was evaporated under a vacuum with a rotary evaporator. The remaining lipid film was hydrated with 250 mM ammonium sulfate in a water bath sonicator for 3 min followed by probe-type sonication for 10 min. The suspensions were then serially filtered through polycarbonate membranes (pore sizes 400 and 200 nm) 3 times each to yield dihydroartemisinin liposomes.

To construct dihydroartemisinin plus epirubicin liposomes, above dihydroartemisinin liposomes were dialyzed (12,000–14,000 molecular mass cutoff) against HEPES-buffered saline (25 mM HEPES, 150 mM NaCl) twice for 12 h each. Dihydroartemisinin liposomes were then added to epirubicin hydrochloride (R&D systems, Minneapolis, MN, USA). After mixing, the suspensions were incubated at 40 °C in a water bath and intermittently shaken for 30 min to produce liposomes containing both drugs.

To construct epirubicin liposomes, the same method for synthesizing the dihydroartemisinin and epirubicin liposomes was used, except that there was no addition of dihydroartemisinin.

The mean particle sizes, polydispersity indexes (PDI), and zeta potential values of all liposomes were measured using the Nano Series Zenith 4003 Zetasizer (Malvern Instruments Ltd., Malvern, UK).

### 2.2. Cell Culture

Human breast cancer cell lines (*MDA-MB-435S*, *MDA-MB-231*, and *MCF-7*) were purchased from the Institute of Basic Medical Science, Chinese Academy of Medical Science (Beijing, China) and used below passage 10. *MDA-MB-435S* and *MDA-MB-231* cells were grown in Leibovitz’s L15 medium (Macgene Biotech Co., Ltd., Beijing, China) supplemented with 10% fetal bovine serum (FBS, PAN, Adenbach, Germany) in a 37 °C humidified incubator. *MCF-7* cells were cultured in RPMI 1640 (Macgene Biotech Co., Ltd., Beijing, China) supplemented with 10% FBS in a humidified incubator at 37 °C under 5% CO_2_.

### 2.3. Cellular Uptake and Mitochondrial Co-Localization

To measure cellular uptake of liposomes, cancer cells were seeded at a density of 2 × 10^5^ cells/well in 6-well culture plates for 24 h. The cells were treated with free epirubicin (10 μM), epirubicin liposomes (10 μM), or dihydroartemisinin plus epirubicin liposomes (20 μM dihydroartemisinin, 10 μM epirubicin) for 8, 12, or 16 h. Culture medium was used as a blank control. After incubation, cells were harvested and washed twice with cold phosphate-buffered saline (PBS, pH 7.4, 137 mM NaCl, 2.7 mM KCl, 8 mM Na_2_HPO_4_, and 2 mM KH_2_PO_4_). Cells (1 × 10^4^) were collected and fluorescence intensity was measured using a flow cytometer (FACSCalibur, Becton Dickinson, Franklin Lakes, NJ, USA) according to the manufacturer’s instructions. Each assay was repeated in triplicate.

Coumarin was used as a fluorescent probe to assess mitochondrial co-localization. Briefly, cancer cells were seeded into chambered cover slides at a density of 2 × 10^5^ cells/dish. After 24 h of incubation, cells were treated with free coumarin (1 μM), coumarin liposomes (1 μM), or coumarin plus dihydroartemisinin liposomes (1 μM coumarin, 10 μM dihydroartemisinin) for 16 h. Culture medium was used as a blank control. The cells were then washed with PBS, and mitochondria were stained with Mitotracker Deep Red (20 nM, Life Technologies Corporation, Carlsbad, NM, USA) at 37 °C for 30 min. Nuclei were stained with Hoechst 33342 (5 μg/mL) for 10 min. Finally, the cells were imaged using confocal microscopy (Leica, Oskar-Barnack, Germany). Each assay was repeated in triplicate.

### 2.4. Induction of Autophagy

Autophagy was quantified using a monodansylcadaverine (MDC) staining kit (Leagene, China). Cancer cells were seeded into 6-well culture plates at a density of 2 × 10^5^ cells/well for 24 h at 37 °C. Cells were treated with free dihydroartemisinin (10 μM), free epirubicin (5 μM), free dihydroartemisinin plus free epirubicin (10 μM dihydroartemisinin, 5 μM epirubicin), dihydroartemisinin liposomes (10 μM), epirubicin liposomes (5 μM), or dihydroartemisinin plus epirubicin liposomes (10 μM dihydroartemisinin, 5 μM epirubicin). The molar ratio of dihydroartemisinin to epirubicin was 2:1 in the liposomal formulation when they were simultaneously incorporated into one liposomal vesicle. Culture medium was used as a blank control. After incubation for 10 h, the cells were washed with a buffer provided in the kit, and incubated with the MDC stain at room temperature for 15 min in the dark. Subsequently, the cells were washed and suspended in a collection buffer provided in the kit. Both buffers were properly diluted according to the manufacturer’s instructions. Extent of autophagy in the cancer cells was determined by the fluorescence intensity measured on a flow cytometer (FACSCalibur, Becton Dickinson, Franklin Lakes, NJ, USA).

Direct observation of autophagy was performed on cancer cells seeded into 6-well culture plates for 24 h, followed by treatment with the same drug formulations as above. After MDC staining as described previously, cell suspensions were dripped onto a glass slide and imaged using confocal microscopy (Nikon, Tokyo, Japan).

### 2.5. Induction of Apoptosis

Apoptosis was assayed using an Annexin V-kFluor647 staining kit (Nanjing Keygen Biotech. Co., Ltd., Nanjing, China) and the nuclear dye SYTOX Green (Nanjing Keygen Biotech. Co., Ltd., Nanjing, China). Cancer cells were seeded at a density of 2 × 10^5^ cells/well in 6-well culture plates for 24 h at 37 °C. Cells were treated with free dihydroartemisinin (10 μM), free epirubicin (5 μM), free dihydroartemisinin plus free epirubicin (10 μM dihydroartemisinin, 5 μM epirubicin), dihydroartemisinin liposomes (10 μM), epirubicin liposomes (5 μM), or dihydroartemisinin plus epirubicin liposomes (10 μM dihydroartemisinin, 5 μM epirubicin). Culture medium was used as a blank control. After incubation for 12 h, the cells were collected, resuspended in the binding buffer provided in the kit, and 10 μL Annexin V-kFluor647 was added. The suspensions were incubated at room temperature for 15 min in the dark. Finally, 5 µL of SYTOX Green was added to each sample for 5 min before analysis on a flow cytometer (Gallios, Beckman Coulter, Brea, CA, USA). Each assay was repeated in triplicate.

### 2.6. Mechanisms of Autophagy and Apoptosis

To evaluate mechanisms of autophagy and apoptosis, cells were seeded into 96-well plates and incubated for 24 h. Cells were then treated with free dihydroartemisinin (10 μM), free epirubicin (5 μM), free dihydroartemisinin plus free epirubicin (10 μM dihydroartemisinin, 5 μM epirubicin), dihydroartemisinin liposomes (10 μM), epirubicin liposomes (5 μM), or dihydroartemisinin plus epirubicin liposomes (10 μM dihydroartemisinin, 5 μM epirubicin). Culture medium was used as a blank control. After incubation for 12 h, cells were fixed with 4% formaldehyde for 15 min, permeabilized with 0.5% Triton X-100 for 15 min, and blocked with 10% goat serum supplemented with 0.3 M glycine for 2 h at room temperature. Cells were then incubated at 4 °C overnight with the following primary antibodies: anti-Beclin 1 (Bioss, Beijing, China), anti-LC3B (Beyotime, Shanghai, China), anti-Bcl-2, anti-Bax, anti-Caspase 3, or anti-Caspase 9 (Sangon, Shanghai, China). Cells were then incubated with the appropriate secondary antibody conjugated to Alexa Fluor-488 (OriGene Local Agent, Beijing, China) at room temperature for 2 h. Both primary and secondary antibodies were diluted according to the manufacturer’s instructions. Nuclei were stained with Hoechst 33342 (5 μg/mL) for 10 min at room temperature. The fluorescence intensity of each well was measured using the Operetta High-Content Screening System (Perkin Elmer, Waltham, MA, USA) and calculated with the Columbus system (Waltham, MA, USA).

### 2.7. Morphological Changes

Changes in cell morphology following drug treatments were assessed by seeding cancer cells into a 96-well plate at a density of 6 × 10^3^ cells/well and culturing for 24 h. The cells were treated for 12 h with free dihydroartemisinin (10 μM), free epirubicin (5 μM), free dihydroartemisinin plus free epirubicin (10 μM dihydroartemisinin, 5 μM epirubicin), dihydroartemisinin liposomes (10 μM), epirubicin liposomes (5 μM), or dihydroartemisinin plus epirubicin liposomes (10 μM dihydroartemisinin, 5 μM epirubicin). Culture medium was used as a blank control. After incubation, cells were harvested and washed twice with cold PBS (pH 7.4), and mitochondria were stained with Mitotracker Deep Red (20 nM, Life Technologies Corporation, Carlsbad, NM, USA) at 37 °C for 30 min. Afterwards, nuclei were stained with Hoechst 33342 (5 μg/mL) for 10 min. Finally, cells were imaged using the Operetta High-Content Screening System (Perkin Elmer, Waltham, MA, USA), and calculations were performed using the Columbus system (Waltham, MA, USA).

### 2.8. Inhibitory Effects In Vitro

Cancer cells were seeded into a 96-well plate at a density of 6 × 10^3^ cells/well and cultured for 24 h. Fresh medium containing the free drug or drug formulation was added to each well, including free epirubicin (0–2.5 µM), a fixed concentration of free dihydroartemisinin (1, 2.5, or 5 µM) plus free epirubicin (0–2.5 µM), dihydroartemisinin liposomes (0–5 µM), epirubicin liposomes (0–5 µM), or dihydroartemisinin plus epirubicin liposomes (0–10 µM for dihydroartemisinin, 0–5 µM for epirubicin). Culture medium was used as a blank control. After incubation for 48 h, cytotoxicity was determined by sulforhodamine B staining assay based on absorbance measurements at a wavelength of 540 nm using a microplate reader (Infinite F50, Tecan Group Ltd., Shanghai, China). The survival rate was calculated using the following formula: Cell survival % = (A_540nm_ for the treated cells/A_540nm_ for the control cells) × 100%, where A_540nm_ is the absorbance value.

### 2.9. Anticancer Efficacy in Cancer-Bearing Mice

Female BALB/c nude mice (weighing 15–17 g) were obtained from Peking University Health Science Center. All animal experiments were performed in accordance with the principles of care and use of laboratory animals, and were approved by the Institutional Animal Care and Use Committee of Peking University. Briefly, approximately 1 × 10^7^ *MDA-MB-435S* cells were resuspended in 200 μL of serum-free medium and injected subcutaneously into the right flanks of nude mice. When tumors reached 100–150 mm^3^ in volume, mice were randomly divided into five treatment groups (*N* = 5 per group). At the 10th, 12th, 14th, and 16th day after inoculation, physiological saline, free epirubicin (4 mg/kg), dihydroartemisinin liposomes (4 mg/kg), epirubicin liposomes (4 mg/kg), or dihydroartemisinin plus epirubicin liposomes (4 mg/kg for both drugs) were administered to mice via tail vein injection. The mice were then monitored and the tumor volume was calculated according to the following formula: the tumor volume (mm^3^) on the *n*th day = length × width^2^/2. A routine blood analysis of the mice was also conducted using a MEK-6318K Hematology Analyzer (Nihon Kohden, Tokyo, Japan).

### 2.10. In Vivo Imaging

Noninvasive optical imaging systems were used to observe the real-time distribution and tumor accumulation of DiR (1,10-dioctadecyl-3,3,30,30 tetramethyl indotricarbocyanine iodide) plus dihydroartemisinin liposomes in breast cancer-bearing xenografts in BALB/c nude mice. DiR was used as fluorescence probe to replace epirubicin for indicating the distribution of nanostructured epirubicin plus dihydroartemisinin liposomes. Female mice were divided into four groups (*N* = 3 per group). After inoculation with *MDA-MB-435S* cells at day 19, the tumor volumes reached approximately 300 mm^3^. These mice were then injected with physiological saline, free DiR, DiR liposomes, or DiR plus dihydroartemisinin liposomes via the tail vein. Mice were scanned at 0.5, 1, 3, 6, 9, 12, and 24 h using an IVIS Spectrum system (Xenogen Corporation, Alameda, CA, USA) under anesthesia with isoflurane.

To visualize the distribution status in tumors and in major organs, the tumor-bearing mice were sacrificed at 36 h after drug administration, and the tumor masses, heart, liver, spleen, lungs, and kidneys were immediately removed. The fluorescent signal intensities in different tissues were imaged and analyzed.

### 2.11. Statistical Analysis

Data are presented as the means ± standard deviation. One-way analysis of variance was used to determine significance among the groups. Post hoc tests with a Bonferroni correction were used to make comparisons between individual groups. *p* < 0.05 was considered statistically significant.

## 3. Results

### 3.1. Characterization of Liposome Formulations

The various liposome formulations were characterized before performing experiments on the cells and mice (Table S1). To summarize, the average particle sizes of the liposomes ranged from 90 to 100 nm with a narrow PDI (approximately 0.2). Zeta potential values were approximately electrically neutral. The encapsulation efficiencies of both dihydroartemisinin and epirubicin were about 90% for all liposomes.

### 3.2. Cellular Uptake and Co-Localization Effect

**Figure 1:**
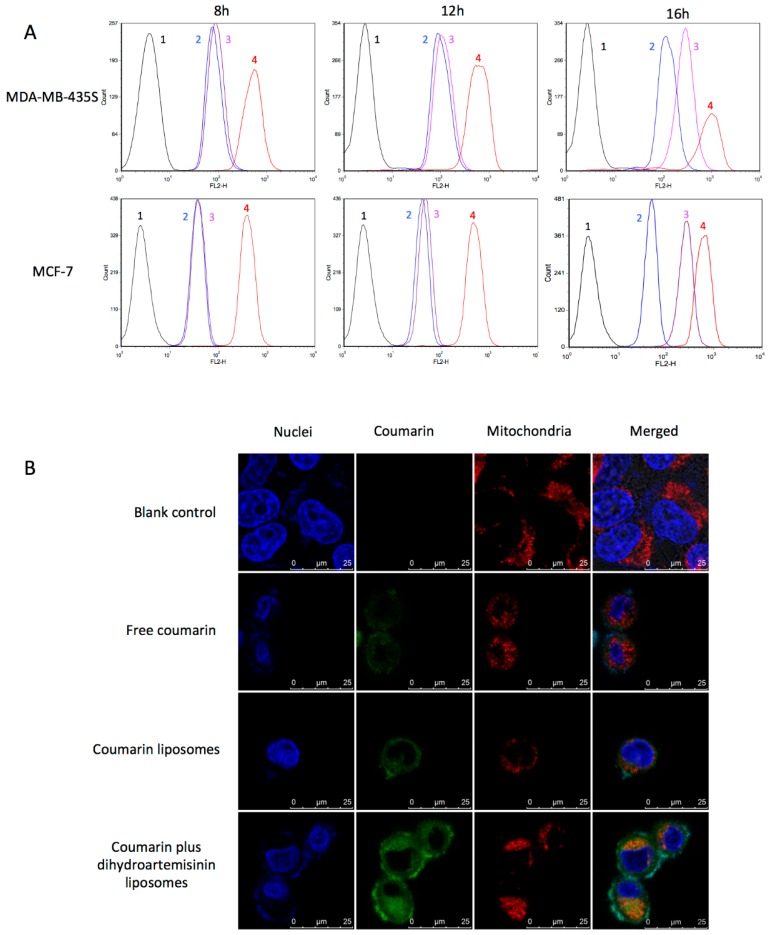
Cellular uptake and co-localization effects in breast cancer cells. (**A**) drug uptake measured by flow cytometry in breast cancer *MDA-MB-435S* and *MCF-7* cells; (**B**) co-localization effect of fluorescent indicator coumarin with mitochondria observed by confocal microscope in breast cancer *MCF-7* cells (scale bar = 25 μm). 1, blank medium; 2, epirubicin liposomes; 3, dihydroartemisinin plus epirubicin liposomes; 4, free epirubicin.

Cellular uptake was indicated by the fluorescence intensity in *MDA-MB-435S* cells after treatment with varying drug formulations (Figure 1A). Results showed that the rank of cellular uptake was free epirubicin > dihydroartemisinin plus epirubicin liposomes > epirubicin liposomes > blank control after treatment at 16 h. Among treatment groups, the cellular uptakes between liposomes exhibited less distinction patterns at 8 h or 12h.

The fluorescence probe coumarin was used to label the liposomes for evaluating the co-localization effect with mitochondria (Figure 1B). In the confocal images, coumarin exhibited green fluorescence, mitochondria exhibited red fluorescence, whereas the nuclei were stained in blue. Bright yellow fluorescence was a composite image of green and red fluorescence, and used to indicate the co-localization effect of drug formulations with mitochondria in *MCF-7* cells. Results demonstrated that the coumarin plus dihydroartemisinin liposomes were co-localized with mitochondria, and exhibited the highest green fluorescence intensity in the cells as compared to other drug formulations.

### 3.3. Induction of Autophagy

**Figure 2:**
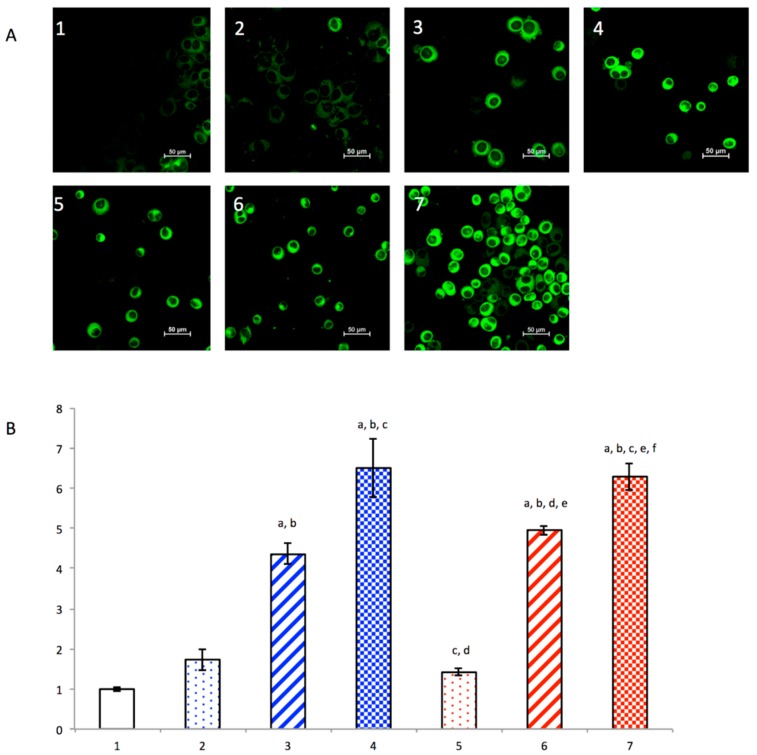
Autophagy induced in breast cancer breast cancer MDA-MB-435S cells. (**A**) qualitative confocal observation on the induced autophagy of breast cancer after processing with monodansylcadaverine kit; (**B**) quantitative flow cytometric estimation on the induced autophagy of breast cancer cells after processing with monodansylcadaverine. 1, blank control; 2, free dihydroartemisinin; 3, free epirubicin; 4, free dihydroartemisinin plus epirubicin (mole ratio = 2:1); 5, dihydroartemisinin liposomes; 6, epirubicin liposomes; 7, dihydroartemisinin plus epirubicin liposomes. Data are presented as the mean ± standard deviation (*n* = 3). *p* < 0.05; a, vs. 1; b, vs. 2; c, vs. 3; d, vs. 4; e, vs. 5; f, vs. 6.

Induction of autophagy in *MDA-MB-435S* cells was evaluated by the MDC staining assay (Figure 2A). Using confocal microscopy, the MDC dye (green) was observed to enter autophagosomes. Cells treated with dihydroartemisinin plus epirubicin liposomes or with free dihydroartemisinin plus free epirubicin had stronger fluorescence intensity compared to other drug groups. The autophagy ratios for cancer cells were also measured by flow cytometry (Figure 2B). The results showed that the rank of autophagy ratios was free dihydroartemisinin plus free epirubicin (6.50 ± 0.72) ≈ dihydroartemisinin plus epirubicin liposomes (6.28 ± 0.33) > epirubicin liposomes (4.96 ± 0.12) > free epirubicin (4.35 ± 0.26) > free dihydroartemisinin (1.73 ± 0.27) > dihydroartemisinin liposomes (1.41 ± 0.07) > blank medium (1.00 ± 0.06).

### 3.4. Induction of Apoptosis

**Figure 3:**
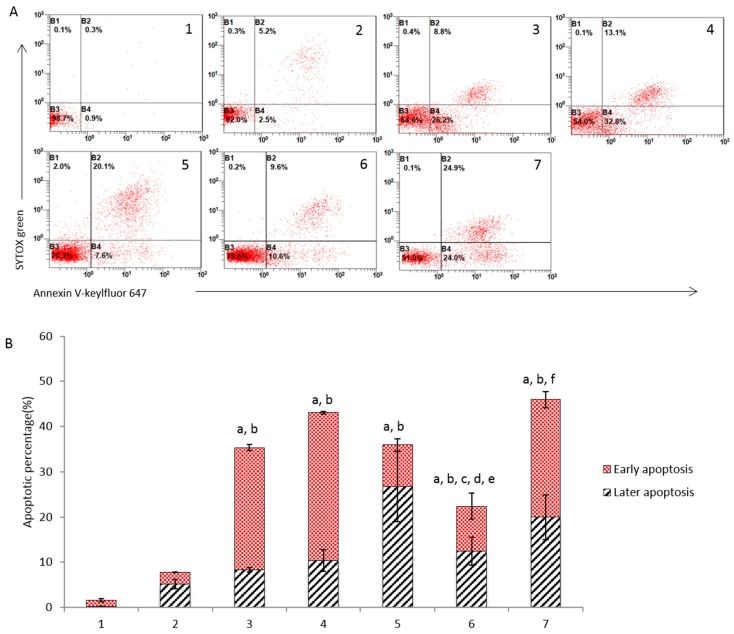
Apoptosis induced in breast cancer MDA-MB-435S cells. (**A**) Flow cytometry plot of breast cancer cells after treatment with varying formulations; (**B**) apoptotic percentage in breast cancer cells after the treatment. 1, blank control; 2, free dihydroartemisinin; 3, free epirubicin; 4, free dihydroartemisinin plus epirubicin (mole ratio = 2:1); 5, dihydroartemisinin liposomes; 6, epirubicin liposomes; 7, dihydroartemisinin plus epirubicin liposomes. Data are presented as the mean ± standard deviation (*n* = 3). *p* < 0.05; a, vs. 1; b, vs. 2; c, vs. 3; d, vs. 4; e, vs. 5; f, vs. 6.

Induction of apoptosis in MDA-MB-435S cells was evaluated by flow cytometry (Figure 3). The results showed that the rank of apoptotic percentage in breast cancer cells was dihydroartemisinin plus epirubicin liposomes (45.9 ± 3.8) ≥ free dihydroartemisinin plus free epirubicin (43.1 ± 2.5) > dihydroartemisinin liposomes (35.9 ± 8.8) > free epirubicin (35.3 ± 0.3) > epirubicin liposomes (22.4 ± 5.1) > free dihydroartemisinin (7.7 ± 1.0) > blank medium (1.5 ± 0.4) (Figure 3B). No significant difference was observed between free drug and the corresponding liposomes (e.g., “free epirubicin” vs. “epirubicin liposomes”; “free dihydroartemisinin plus free epirubicin” vs. dihydroartemisinin plus epirubicin liposomes), demonstrating that the nanostructured dihydroartemisinin plus epirubicin liposomes significantly enhancing the apoptotic effects although the liposome vesicle itself did not alter the apoptosis-inducing capability of free drugs.

### 3.5. Mechanism of Autophagy and Apoptosis

**Figure 4:**
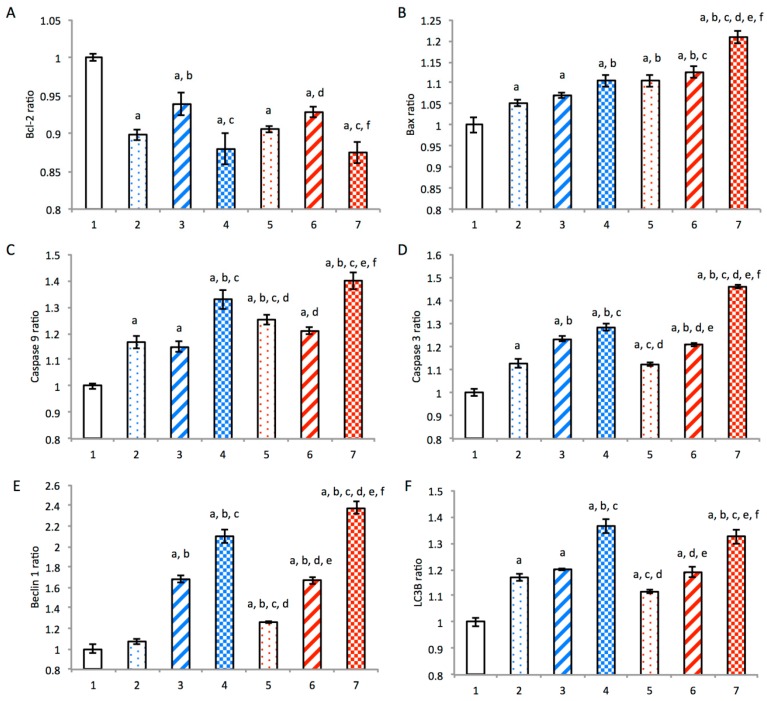
Mechanisms of autophagy and apoptosis in breast cancer MDA-MB-435S cells. 1, blank control; 2, free dihydroartemisinin; 3, free epirubicin; 4, free dihydroartemisinin plus epirubicin (mole ratio = 2:1); 5, dihydroartemisinin liposomes; 6, epirubicin liposomes; 7, dihydroartemisinin plus epirubicin liposomes. Data are presented as the mean ± standard deviation (*n* = 3). *p* < 0.05; a, vs. 1; b, vs. 2; c, vs. 3; d, vs. 4; e, vs. 5; f, vs. 6.

The mechanisms of autophagy and apoptosis were evaluated by measuring autophagy- and apoptosis-related proteins in *MDA-MB-436S* cells (Figure 4). After incubation with the blank medium, free dihydroartemisinin, free epirubicin, or free dihydroartemisinin plus free epirubicin, the expression levels of the anti-apoptotic protein Bcl-2, the pro-apoptotic protein Bax, the apoptotic enzymes Caspase-9 and -3, and autophagy-related proteins Beclin 1 and LC3B were assessed. The activity ratios of Bcl-2 were 1.00 ± 0.01, 0.89 ± 0.01, 0.93 ± 0.01, 0.88 ± 0.02, 0.91 ± 0.00, 0.93 ± 0.01, and 0.87 ± 0.01; the activity ratios of Bax were 1.00 ± 0.02, 1.05 ± 0.01, 1.07 ± 0.01, 1.11 ± 0.01, 1.11 ± 0.01, 1.13 ± 0.01, and 1.21 ± 0.01; the activity ratios of Caspase-9 were 1.00 ± 0.01, 1.17 ± 0.03, 1.15 ± 0.02, 1.33 ± 0.04, 1.25 ± 0.02, 1.21 ± 0.01, and 1.40 ± 0.03; the activity ratios of Caspase-3 were 1.00 ± 0.01, 1.13 ± 0.02, 1.23 ± 0.01, 1.28 ± 0.01, 1.12 ± 0.01, 1.21 ± 0.01, and 1.46 ± 0.01; the activity ratios of Beclin 1 were 1.00 ± 0.04, 1.07 ± 0.03, 1.68 ± 0.04, 2.10 ± 0.06, 1.26 ± 0.01, 1.67 ± 0.04, and 2.37 ± 0.06; the activity ratios of LC3B were 1.00 ± 0.02, 1.17 ± 0.01, 1.20 ± 0.00, 1.36 ± 0.03, 1.12 ± 0.01, 1.19 ± 0.02, and 1.33 ± 0.03, respectively. Compared to control groups, dihydroartemisinin plus epirubicin liposomes significantly increased the expressions of Caspase-9, Caspase-3, Bax, Beclin 1, and LC3B, while evidently suppressing the expression of Bcl-2. In addition, the levels of reactive oxygen species (ROS) in the cells treated with dihydroartemisinin plus epirubicin liposomes were significantly higher compared to the controls (Figure S1).

### 3.6. Morphological Changes

Morphological changes in the cells treated with the various formulations were evaluated with a high-content screening system to visualize the mitochondria (red) and nuclei (blue) (Figure S2). Both qualitative (Figure S2A) and quantitative (Figure S2B) measurements showed that mitochondria were disrupted, and they displayed an obvious decrease in fluorescence after drug treatments. Changes in the spherical morphology of the nuclei were also observed (Figure S2C). Specifically, after treatment with the blank medium, free dihydroartemisinin, free epirubicin, free dihydroartemisinin plus free epirubicin, dihydroartemisinin liposomes, epirubicin liposomes, or dihydroartemisinin plus epirubicin liposomes, the fluorescence intensities of the mitochondria were 384.13 ± 7.54, 312.51 ± 8.11, 285.90 ± 11.15, 228.08 ± 8.50, 315.40 ± 6.70, 289.96 ± 14.12, and 219.21 ± 12.86; the nuclear roundness was 0.96 ± 0.00, 0.92 ± 0.01, 0.86 ± 0.01, 0.81 ± 0.01, 0.93 ± 0.01, 0.84 ± 0.01, and 0.80 ± 0.01, respectively. These results indicated that the integrity of the mitochondria and nuclei of the cancer cells were disrupted by treatment with dihydroartemisinin plus epirubicin liposomes, respectively.

### 3.7. Cytotoxic Effects In Vitro

In vitro cytotoxicity was evaluated in the MDA-MB-435S, MDA-MB-231, and MCF-7 cells (Figure S3). Results indicated that a single free epirubicin treatment caused limited cytotoxicity in cancer cells, while co-treatment of free dihydroartemisinin with free epirubicin significantly enhanced this effect in a concentration-dependent manner for dihydroartemisinin (Figure S3A–C). Furthermore, dihydroartemisinin plus epirubicin liposomes exhibited the strongest cytotoxicity at various dosages. In addition, dihydroartemisinin liposomes displayed a moderately cytotoxic effect (Figure S3D–F).

### 3.8. Anticancer Efficacy and In Vivo Imaging

**Figure 5:**
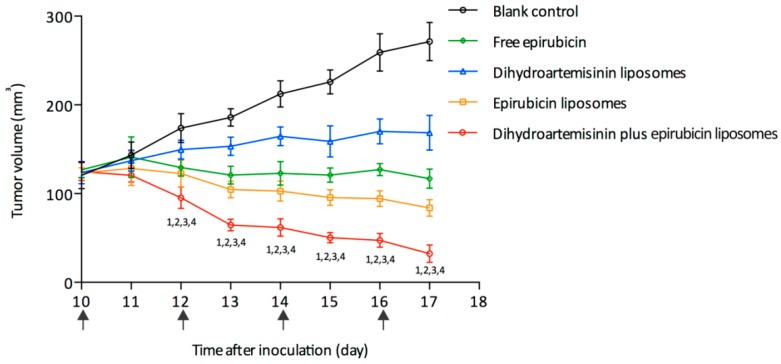
Antitumor efficacy in breast cancer MDA-MB-435S xenografts in nude mice. The arrows indicate the day of drug administration. Data are presented as the mean ± standard deviation (*n* = 5). *p* < 0.05; 1, vs. blank control; 2, vs. free epirubicin; 3, vs. dihydroartemisinin liposomes; 4, vs. epirubicin liposomes.

Anticancer efficacy was evaluated with respect to tumor volume in xenografts of MDA-MB-435S cells in mice (Figure 5). After treatment with the various formulations, the ranking for tumor volumes was as follows: physiological saline > dihydroartemisinin liposomes > free epirubicin > epirubicin liposomes > dihydroartemisinin plus epirubicin liposomes. These results indicated that the dihydroartemisinin plus epirubicin liposomes had the strongest overall anticancer efficacy. In addition, the body weights of tumor-bearing mice did not differ among the experimental groups (Figure S4). After treatment with free epirubicin, white blood cells of the mice significantly decreased. In contrast, liposomal drug formulations did not cause obvious changes in blood indices (Figure S5, Table S2).

Real-time imaging of fluorescently labeled liposomes was also performed on the xenografts of MDA-MB-435S cells in these mice (Figure S6A). After treatment with free DiR, the fluorescent signal rapidly accumulated in the liver. In contrast, after treatment with DiR plus dihydroartemisinin liposomes or DiR liposomes, the fluorescent signal remained predominantly in the blood system and tumor tissues. Ex vivo imaging similarly showed that the fluorescent signal for DiR plus dihydroartemisinin liposomes remained in the tumor tissues, while those of control formulations were only weakly visible or invisible (Figure S6B). In addition, the fluorescent signal was detected in the livers and spleens of these mice after treatment with all DiR formulations.

## 4. Discussion

In a comprehensive treatment strategy for breast cancer, chemotherapy is an important tool used to eliminate cancer cells. However, breast cancer is a typically heterogeneous malignancy, which leads to poor clinical outcomes. Most current treatment strategies rely on inducing necrosis and type I programmed cell death (apoptosis). Nevertheless, this approach is often inefficient at eradicating heterogeneous cancer cells. In this study, we propose a new strategy for overcoming this clinical problem by initiating cross-talk between autophagy and apoptosis in cancer cells, which may help increase the efficacy of heterogeneous breast cancer therapies.

In the dihydroartemisinin plus epirubicin liposomes, dihydroartemisinin incorporated into the lipid bilayer, while epirubicin was encapsulated in the inner vesicle of the liposome. The liposomes were coated with DSPE-PEG_2000_ and exhibited a homogeneously distributed nanoscale particle size (Table S1). This prolonged the circulation of the drugs in the blood stream by avoiding rapid clearance by the reticuloendothelial system [30]. Thus, the drugs accumulation in tumor tissues as a result of enhanced permeability and retention effects [31,32,33]. Meanwhile, the liposomal drug formulation also reduced the systemic cytotoxicity of epirubicin by decreasing concentrations in healthy tissues. This is because anthracyclines have cardiotoxic side effects that can lead to myocardial damage. As a typical example of anthracyclines, the cardiotoxicity of doxorubicin could be reduced by liposomal encapsulation [34]. Epirubicin has the same mechanism of myocardial damage as doxorubicin, therefore, the liposome encapsulation has the potential to reduce the distribution of epirubicin in heart tissue, thereby reducing cardiotoxicity.

The evaluation of cellular uptake of liposomes revealed that both dihydroartemisinin and epirubicin were efficiently internalized by cancer cells. Moreover, dihydroartemisinin significantly enhanced the co-uptake of epirubicin (Figure 1). This effect was time-dependent, and could be due to the disruption of the cell membrane of the cancer cells exposed to dihydroartemisinin.

Under normal conditions, autophagy allows the orderly degradation and recycling of unnecessary or dysfunctional cellular components to maintain cytoplasmic homeostasis. In the case of a diseased cell, moderate autophagy facilitates an adaptive response to stress that promotes cancer cell survival and metastasis. However, excessive autophagy can ultimately result in type II programmed death of cancer cells [35]. In this study, we found that dihydroartemisinin induced excessive autophagy in cancer cells, and the co-treatment of dihydroartemisinin and epirubicin in liposomes caused significant autophagy that resulted in type II programmed death of breast cancer cells (Figure 2).

In the initiation of autophagy, dihydroartemisinin alone and dihydroartemisinin plus epirubicin released from liposomes activated Beclin 1, which played an essential role in autophagosome formation as a core component of the Class III phosphatidylinositol 3-kinase (PI3K) complex. The Class III PI3K complex utilized PtdIns as a substrate to generate PtdIns3P, which mediated the recruitment of other autophagy proteins onto the pre-autophagosomal membrane [36]. This resulted in excessive autophagy. These effects were demonstrated using the enhanced expression of LC3B, which was the most commonly used autophagy-related marker [37].

Apoptosis is a highly regulated process of cell death and helps maintain survival/death homeostasis in multicellular organisms [38]. It can be initiated through two signaling pathways: the intrinsic mitochondrial pathway and the extrinsic death receptor signaling pathway [39]. Because multidrug resistance exists in heterogeneous cancer cells, the induction of apoptosis is a common approach for improving the efficacy of chemotherapy treatments. In this study, we found that the dihydroartemisinin plus epirubicin formulation significantly enhanced apoptosis in breast cancer cells (Figure 3).

In the initiation of apoptosis, the liposomal release of dihydroartemisinin alone and the combination of dihydroartemisinin plus epirubicin activated the pro-apoptotic protein Bax, which was attached into mitochondria to permeabilize the mitochondrial outer membrane. This process initiated a cascade of apoptotic reactions by activating the upstream initiator Caspase-9 and the downstream effector Caspase-3. This ultimately led to type I programmed death in the cancer cells.

A study of the literature indicates that ROS mediates the mitochondrial permeability transition and increases expression of apoptosis-related proteins, such as Bax and Caspase-9. Therefore, in our study, the generated ROS might be involved in damaging the mitochondrial membrane, upregulating Bax, and activating Caspase-9, thereby promoting apoptosis via the mitochondrial pathway [40].

**Figure 6:**
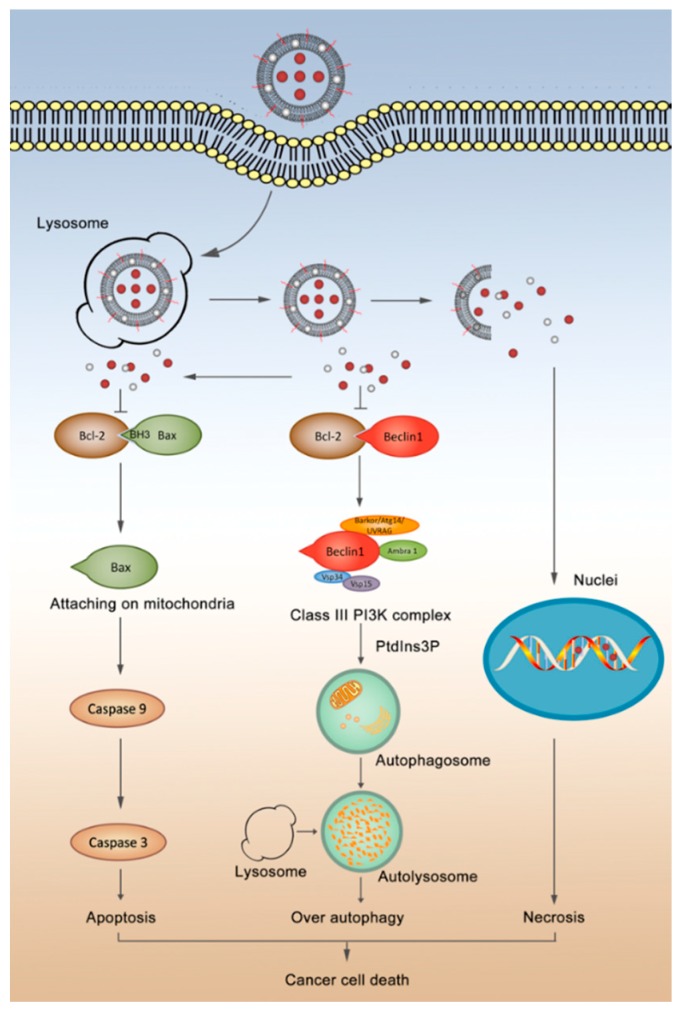
Illustration of heterogeneous cancer death induced by dihydroartemisinin plus epirubicin liposomes through autophagy, apoptosis and necrosis. The nanostructured dihydroartemisinin plus epirubicin liposomes were internalized by cancer cells, escaped from the lysosomes, and existed in two states: the raptured and intact liposomes. Drugs, which either are from the raptured liposomes or are released from the intact liposomes, come into play actions: **1**, dihydroartemisinin interacts with the BH3 domain of Bcl-2, thus suppressing the binding of Bcl-2 with Beclin 1, activating the class III phosphatidylinositol 3-kinase (PI3K) complex to produce phosphatidylinositol 3-phosphate (PtdIns3P) in promoting the excessive autophagy. **2**, dihydroartemisinin suppresses the binding of Bcl-2 with Bax, which allows Bax to attach onto mitochondria, to activate upstream caspase 9 and afterwards downstream caspase 3 in initiating a cascade of apoptosis via the mitochondria pathway. **3**, Epirubicin intercalates DNA strands, resulting in necrosis of cancer cells.

The present study also found that dihydroartemisinin induced cross-talk between autophagy and apoptosis. In this process, Bcl-2 likely interacted with Beclin 1 or Bax, since it was known to be an anti-apoptotic protein that can bind to Bax to block mitochondrial depolarization [41]. Previous studies demonstrated that Bcl-2 prevented autophagy by binding Beclin 1 [42]. In this study, the released dihydroartemisinin or dihydroartemisinin plus epirubicin from liposomes significantly inhibited the activity of Bcl-2, further destabilizing the binding of Bcl-2 with Beclin 1 and Bax. This disaggregation likely resulted in the release of Beclin 1 from the Bcl-2/Beclin 1 complex, and activated the Class III PI3K complex. This would cause excessive autophagy and induce type II programmed death of cancer cells. Meanwhile, this suppression likely promoted the release of Bax from the Bcl-2/Bax complex, allowing Bax to participate in apoptosis. In addition, epirubicin intercalated into DNA, which could have caused the observed necrosis of cancer cells (Figure 4 and Figure 6). Morphological changes in the mitochondria and nuclei (Figure S2) provided additional evidence that dihydroartemisinin or dihydroartemisinin plus epirubicin liposomes destroyed mitochondria and disrupted the integrity of DNA, which also could have contributed to the observed apoptosis and necrosis.

In vitro (Figure S3) and in vivo (Figure 5) experiments showed that dihydroartemisinin alone was moderately cytotoxic in cancer cells, while dihydroartemisinin plus epirubicin liposomes exhibited a robust cytotoxic effect. In vivo imaging also demonstrated that the liposomal formulation allowed the drugs to become significantly enriched in cancer tissues.

## 5. Conclusions

The present study reveals that dihydroartemisinin potentiates the efficacy of epirubicin-based treatment of heterogeneous breast cancer through the induction of autophagy and apoptosis. Dihydroartemisinin inhibits the activity of Bcl-2, which releases active Beclin 1 and Bax from their respective complexes. This results in a synergy between autophagy and apoptosis. The release of Bax activates apoptosis, leading to type I programmed death. Meanwhile, the release of Beclin 1 initiates excessive autophagy, which induces type II programmed death in cancer cells. The combinational use of dihydroartemisinin with epirubicin potentiates these effects, thereby enhancing overall cytotoxicity; namely, cancer cells can be killed by necrosis which is primarily caused by DNA damage with epirubicin, apoptosis and over-autophagy both of which are induced by dihydroartemisinin. The enhanced anticancer efficacy by liposomal formulation of dihydroartemisinin and epirubicin is evidenced in breast cancer-bearing mice. Besides, this effect may be due to the prolonged circulation of drug liposomes in the blood and the concentrated drugs in cancer tissues. Therefore, the nanostructured combinational formulation of dihydroartemisinin plus epirubicin liposomes provides a promising new strategy for the treatment of heterogeneity in breast cancer.

## Supplementary Materials

The following are available online at http://www.mdpi.com/2079-4991/8/10/804/s1, Figure S1: ROS activity ratios in breast cancer MDA-MB-435S cells after treatment with varying drug formulations at 6 h, Figure S2: Morphological changes in mitochondria and nuclei of breast cancer MDA-MB-435S cells, Figure S3: Inhibitory effects to breast cancer cells, Figure S4: Body weight changes of nude mice bearing breast cancer MDA-MB-435S xenografts after treatment with varying formulations, Figure S5: White blood cells in tumor-bearing nude mice after administration of varying drug formulations, Figure S6: Real-time imaging and distribution of liposomes in nude mice bearing breast cancer MDA-MB-435S xenografts, Table S1: Characterization of liposomes, Table S2: Blood examination on tumor-bearing nude mice after administration of drug formulations.
