# Supplementary material for: Nanostructured Dihydroartemisinin Plus Epirubicin Liposomes Enhance Treatment Efficacy of Breast Cancer by Inducing Autophagy and Apoptosis
Source: Nanomaterials (Basel). 2018 Oct 9;8(10):804. doi: 10.3390/nano8100804 (PMC6215314; doi:10.3390/nano8100804)
Supplement: Supplementary file 1 [file nanomaterials-08-00804-s001.pdf]

Supplementary Materials

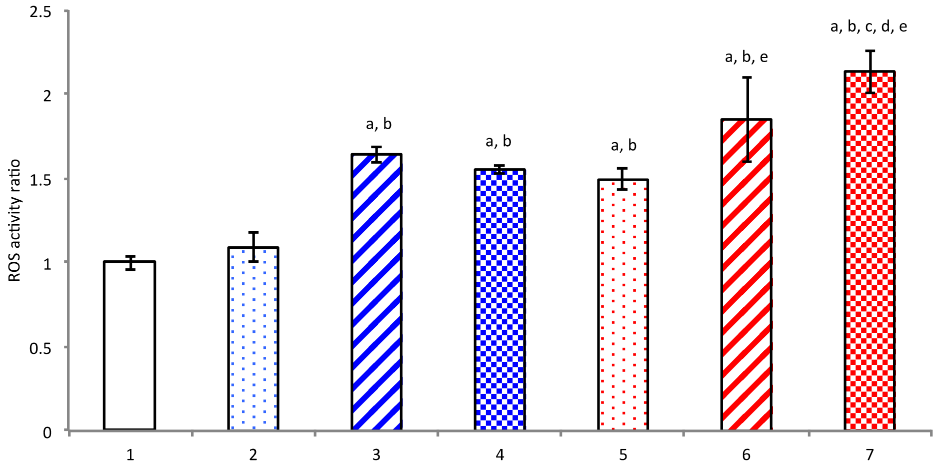

Figure S1. ROS activity ratios in breast cancer MDA-MB-435S cells after treatment with varying drug formulations at 6 h. 1, blank control; 2, free dihydroartemisinin; 3, free epirubicin; 4, free dihydroartemisinin plus epirubicin (mole ratio=2:1); 5, dihydroartemisinin liposomes; 6, epirubicin liposomes; 7, dihydroartemisinin plus epirubicin liposomes. Data are presented as the mean ± standard deviation (n=3). P<0.05; a, vs. 1; b, vs. 2; c, vs. 3; d, vs. 4; e, vs. 5; f, vs. 6.

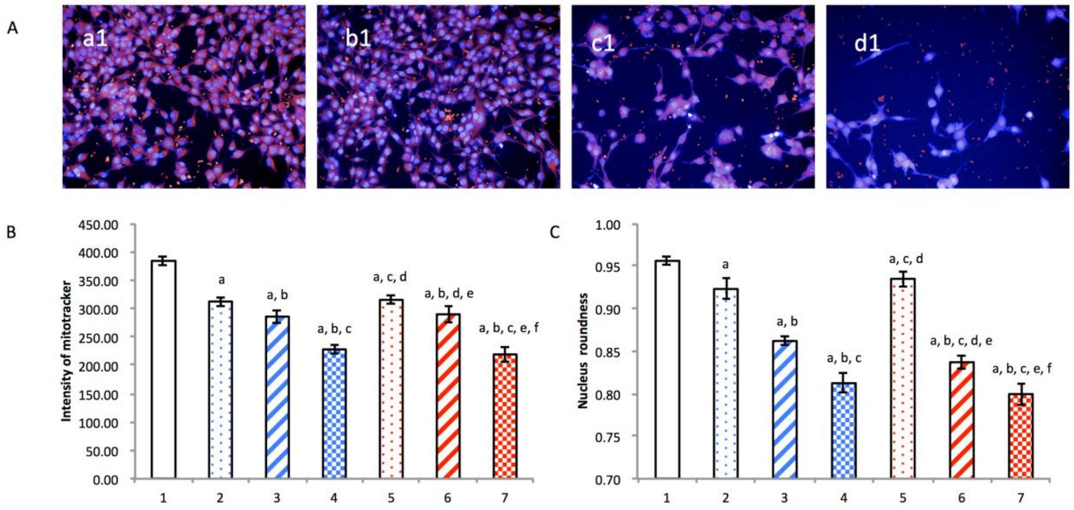

Figure S2. Morphological changes in mitochondria and nuclei of breast cancer MDA-MB-435S cells. (**A**) operetta high content screening observation on cancer cells where mitochondria exhibited red fluorescence and nuclei exhibited blue fluorescence; (**B**) destruction of mitochondria indicated by intensify of mitotracker fluorescence in cancer cells calculated through the Columbus system; (**C**) nucleus roundness of cancer cells calculated through the Columbus system. a1, blank control; b1, dihydroartemisinin liposomes; c1, epirubicin liposomes; d1, dihydroartemisinin plus epirubicin liposomes. 1, blank control; 2, free dihydroartemisinin; 3, free epirubicin; 4, free dihydroartemisinin plus epirubicin (mole ratio=2:1); 5, dihydroartemisinin liposomes; 6, epirubicin liposomes; 7, dihydroartemisinin plus epirubicin liposomes. Data are presented as the mean ± standard deviation (n=3). P<0.05; a, vs. 1; b, vs. 2; c, vs. 3; d, vs. 4; e, vs. 5; f, vs. 6.

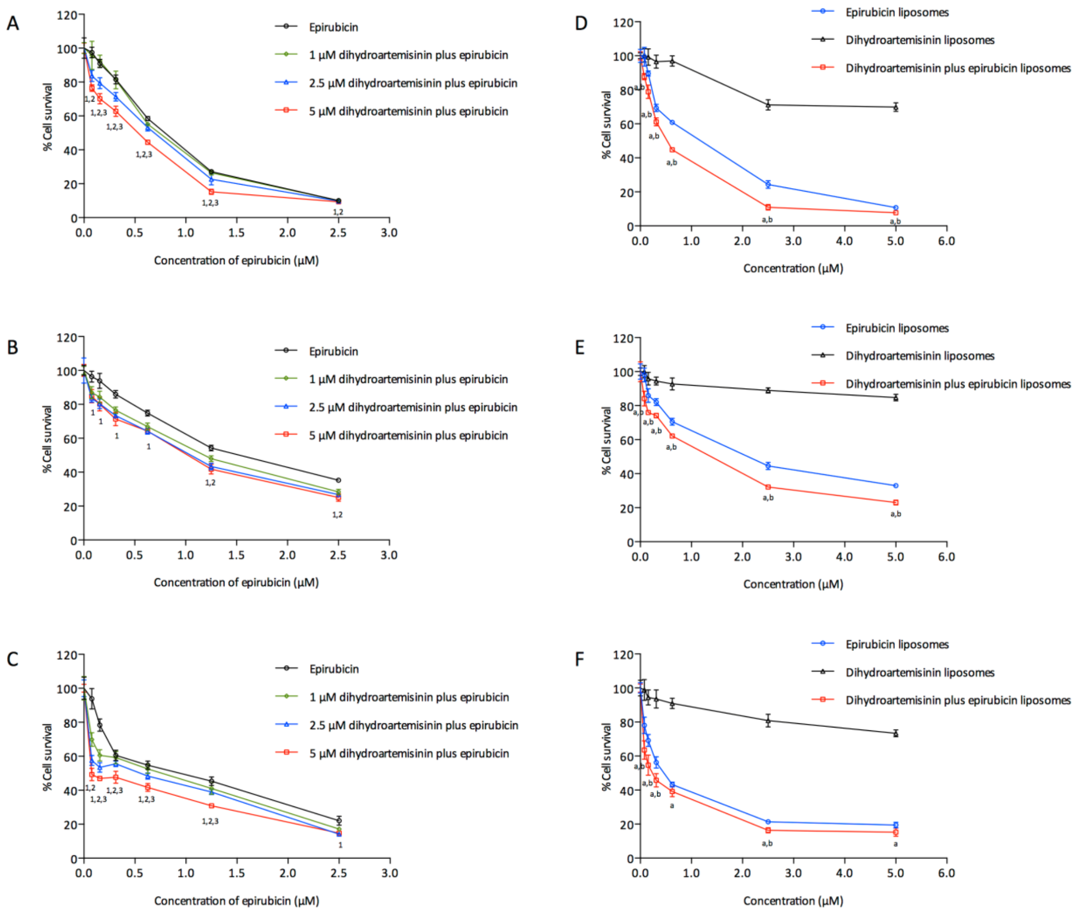

Figure S3. Inhibitory effects to breast cancer cells. (**A**) inhibitory effects to breast cancer MDA-MB-435S cells after treatment with free drugs; (**B**) inhibitory effects to breast cancer MDA-MB-231 cells after treatment with free drugs; (**C**) inhibitory effects to breast cancer MCF-7 cells after treatment with free drugs; (**D**) inhibitory effects to breast cancer MDA-MB-435S cells after treatment with varying liposomal formulations; (**E**) inhibitory effects to breast cancer MDA-MB-231 cells after treatment with varying liposomal formulations; (**F**) inhibitory effects to breast cancer MCF-7 cells after treatment with varying liposomal formulations. Data are presented as the mean ± standard deviation (n=4). $P<0.05$. 1, vs. epirubicin; 2, vs. 1μM dihydroartemisinin plus epirubicin; 3, vs. 2.5μM dihydroartemisinin plus epirubicin. a, vs. dihydroartemisinin liposomes; b, vs. epirubicin liposomes.

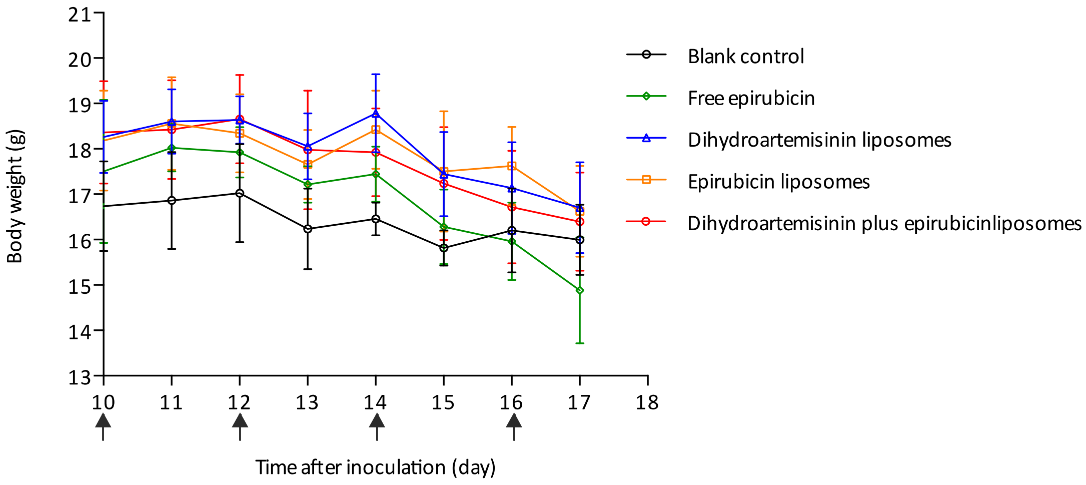

Figure S4. Body weight changes of nude mice bearing breast cancer MDA-MB-435S xenografts after treatment with varying formulations. The arrows indicate the day of drug administration. Data are presented as the mean ± standard deviation (n=5). $P<0.05$; 1, vs. blank control; 2, vs. free epirubicin; 3, vs. dihydroartemisinin liposomes; 4, vs. epirubicin liposomes.

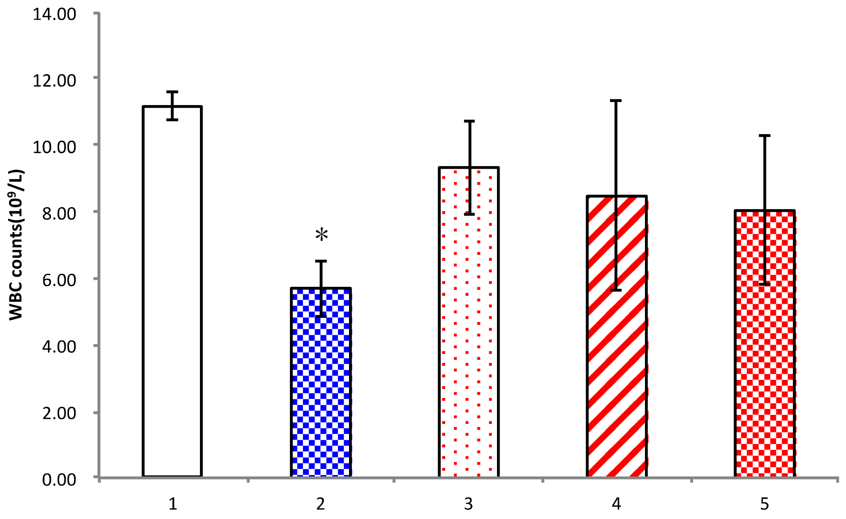

Figure S5. White blood cells in tumor-bearing nude mice after administration of varying drug formulations. 1, blank control; 2, free epirubicin; 3, dihydroartemisinin liposomes; 4, epirubicin liposomes; 5, dihydroartemisinin plus epirubicin liposomes. Data are presented as the mean ± standard deviation (n=3). *$P<0.05$, vs. blank control.

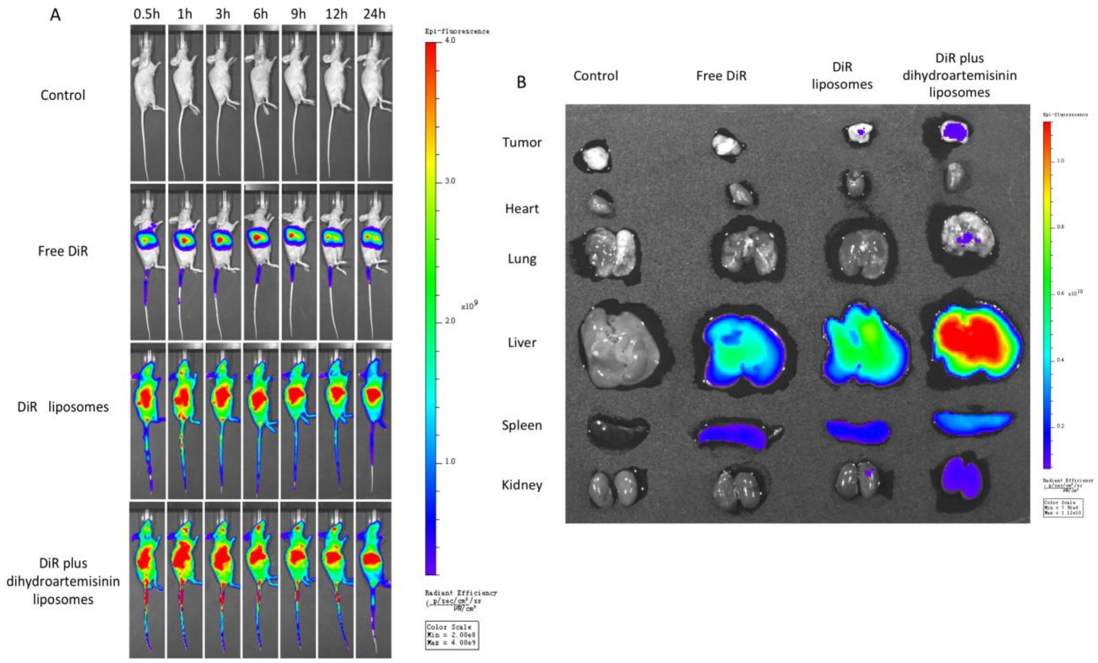

Figure S6. Real-time imaging and distribution of liposomes in nude mice bearing breast cancer MDA-MB-435S xenografts. (**A**) In vivo real-time imaging of the tumor-bearing nude mice. (**B**) Ex vivo optical images of the tumor and normal tissues after the tumor-bearing mice sacrificed at 36 h.

Table S1. Characterization of liposomes

Liposomes	Mean size (nm)	Polydispersity index (PDI)	Zeta potential (mV)	Encapsulation efficiency (%)	
				Dihydroartemisinin	Epirubicin
Blank liposomes	91.61±1.76	0.169±0.008	-0.057±0.091	-	-
Dihydroartemisinin liposomes	93.93±1.22	0.145±0.017	0.467±0.337	93.71±0.39	-
Epirubicin liposomes	94.80±2.97	0.154±0.014	-0.296±0.544	-	96.53±0.61
Dihydroartemisinin plus epirubicin liposomes	95.75±2.78	0.169±0.003	0.254±0.112	91.53±0.20	95.43±0.71

Data are presented as the mean ± standard deviation (n=3).

Table S2. Blood examination on tumor-bearing nude mice after administration of drug formulations

Assay	Saline	Free epirubicin	Dihydroartemisinin liposomes	Epirubicin liposomes	Dihydroartemisinin plus epirubicin liposomes
WBC[a](10^9/L)	11.17±0.4	5.7±0.85*	9.33±1.42	8.47±2.86	8.03±2.24
RBC[b](10^{12}/L)	9.33±0.24	8.91±0.4	9.46±0.49	8.88±1.04	8.24±1.46
HGB[c](g/L)	129±4.58	129±3.46	133.67±5.86	132±11.53	118.67±14.74
HCT[d](%)	38.43±0.86	37.77±0.55	39.63±1.31	38.03±4.11	34.07±4.39
MCV[e](fL)	41.2±1.68	42.43±1.33	41.9±0.89	42.87±0.8	41.63±2.32
MCH[f](pg)	13.83±0.8	14.53±0.83	14.1±0.3	14.9±0.72	14.53±1.01
MCHC[g](g/L)	336±7.81	341.67±10.02	337.33±9.07	347.67±10.21	348.67±8.5
RDW[h](%)	14.93±0.51	14.23±0.38	15.03±0.45	14.4±0.62	15.07±1.07
PLT[i](10^9/L)	774.33±53.91	985.67±251	897.67±210.76	1195.33±353.55	1216.67±199.36
PCT[j](%)	0.31±0.02	0.4±0.1	0.4±0.12	0.52±0.16	0.58±0.11
MPV[k](fL)	4.07±0.06	4.13±0.12	4.5±0.26	4.37±0.21	4.77±0.21*
PDW[l](%)	12.7±0.56	14.27±0.65	14.1±0.72	14.7±1.3	14.4±0.7
LYM[m](10^9/L)	1.17±0.06	0.47±0.15	1.33±0.61	2.83±2.95	0.73±0.35
MID[n](10^9/L)	0.67±0.38	0.3±0.26	1.4±0.92	1.83±1.54	0.43±0.25
GRN[o](10^9/L)	9.33±0.15	4.93±0.93*	6.6±0.53	3.8±1.83*	6.87±1.94

Notes: a, white blood cells; b, red blood cells; c, hemoglobin; d, hematocrit; e, mean corpuscular volume; f, mean corpuscular hemoglobin; g, mean corpuscular hemoglobin concentration; h, red cell distribution width; i, platelets; j, plateletcrit; k, mean platelet volume; l, platelet distribution width; m, lymphocyte; n, intermediate cell; o, neutrophile granulocyte. Data are presented as the mean ± standard deviation (n=3). *$P<0.05$, vs. blank control.
